# Impact of pre-pregnancy body mass index on preeclampsia

**DOI:** 10.3389/fmed.2025.1529966

**Published:** 2025-02-05

**Authors:** Jing Mao, Hanxiang Sun, Qinxin Shen, Chang Zou, Yuanyuan Yang, Qiaoling Du

**Affiliations:** Department of Obstetrics, Shanghai Key Laboratory of Maternal Fetal Medicine, Shanghai Institute of Maternal-Fetal Medicine and Gynecologic Oncology, Shanghai First Maternity and Infant Hospital, School of Medicine, Tongji University, Shanghai, China

**Keywords:** body mass index, underweight, overweight, obese, preeclampsia

## Abstract

**Background:**

It remains unclear how pre-pregnancy body mass index (BMI) affects preeclampsia in the Chinese population, primarily due to insufficient large-scale research on this topic.

**Objective:**

The study aimed to determine the relationship between pre-pregnancy BMI and (severe) preeclampsia in the Chinese population, providing a detailed description of the findings.

**Methods:**

The retrospective study included a total of 75,773 pregnant women registered between 2016 and 2020. These participants were categorized into four groups based on their pre-pregnancy BMI: underweight (BMI < 18.5 kg/m^2^), normal weight (BMI 18.5–<24 kg/m^2^), overweight (BMI 24–<28 kg/m^2^), and obese (BMI ≥ 28 kg/m^2^). The relationship between risks of preeclampsia or severe preeclampsia and pre-pregnancy BMI were further explored, with an evaluation of potential modification by maternal age.

**Results:**

A lower risk of developing preeclampsia was observed in the underweight population, with an OR of 0.604 (95%CI, 0.507–0.719). In contrast, women who were overweight or obese during the pre-pregnancy period demonstrated a significantly higher risk of preeclampsia, with ORs of 2.211 (95%CI, 1.967–2.486) and 3.662 (95%CI, 3.026–4.431), respectively. After adjusting for confounding factors, the elevated risk of preeclampsia persisted, showing ORs of 2.152(95%CI, 1.911–2.425) for the overweight population and 3.493 (95%CI, 2.874–4.245) for those who were obese, while the risk for underweight women remained lower, with an OR of 0.609(95%CI, 0.511–0.727). For severe preeclampsia, the risk was also higher in the overweight and obese participants after adjusting for confounders, demonstrating ORs of 1.652(95%CI, 1.364–2.001) and 2.762(95%CI, 2.014–3.788), respectively. The underweight population exhibited a lower risk of severe preeclampsia, with an OR of 0.720(95%CI, 0.565–0.919). In addition, these risks were not significantly associated with maternal age.

**Conclusion:**

Regardless of adjustment for confounders, underweight women demonstrated a lower risk of preeclampsia, whereas the overweight/obese population exhibited a higher occurrence of both preeclampsia and severe preeclampsia. These associations were not influenced by maternal age.

## Introduction

Preeclampsia, a hypertensive condition unique to pregnancy, is characterized by high blood pressure and either end-organ dysfunction or proteinuria, typically developing after the 20th week of gestation (1). Preeclampsia serves as a primary contributor to maternal and neonatal mortality and morbidity worldwide, leading to serious complications in both the short term and long term (2), including placental abruption, damage to the maternal liver, kidneys, or brain, premature birth, fetal growth restriction, and an increased risk of chronic hypertension and cardiovascular diseases. Additionally, children born to mothers with preeclampsia may face neurodevelopmental issues and chronic health problems. Preeclampsia with severe complications, including hemolysis, elevated liver enzyme levels, and low platelet levels (HELLP syndrome), along with other serious maternal and fetal health issues, can be further classified as severe preeclampsia (3).

Body mass index (BMI) serves as an important indicator for assessing the nutritional status of women. The effect of pre-pregnancy BMI on adverse obstetric outcomes has been well-documented, such as stillbirth, large for gestational age, gestational diabetes, neonatal mortality, and preterm birth (4–7). In addition, increasing amounts of research have indicated that pre-pregnancy BMI is closely related to the risk of preeclampsia, particularly in women classified as obese or overweight (8–10), with this association potentially being even more significant. The pathophysiological mechanisms underlying obesity and preeclampsia share commonalities, such as heightened oxidative stress, an inflammatory environment, vasoconstriction, and endothelial dysfunction (11).

Despite the well-documented connection between pre-pregnancy BMI and preeclampsia, relatively few studies have specifically addressed the effect of pre-pregnancy overweight or obesity on the risk of severe preeclampsia. The majority of these studies are outdated (12–14), highlighting the ongoing need for updated research. Early reports suggested that women with a pre-pregnancy BMI value ranging from 25.0 to 29.9 or ≥ 30.0 exhibited a higher risk of severe preeclampsia (15). However, with the evolution of society, the constitution and nutritional status of the female population have changed, accompanied by increased awareness and shifting perspectives on maternal health during pregnancy (16, 17). Moreover, there are differences in obesity diagnostic criteria across countries (18, 19), further underscoring the urgent need for a contemporary study with a large sample size, specifically focusing on the domestic female population in China.

Given the evidence and potential clinical implications, our study aimed to explore the relationship between BMI before pregnancy and preeclampsia risk, with a specific focus on severe preeclampsia, based on patient data obtained from Shanghai First Maternity and Infant Hospital in China. This research highlights the significance of weight management before conception in preventing preeclampsia, which can help mitigate severe adverse pregnancy outcomes and related complications. Our research aimed to provide precise estimates on the relationship between preeclampsia and pre-pregnancy BMI, particularly in high-risk populations with extreme BMI values, ultimately enhancing maternal and fetal health outcomes.

## Materials and methods

### Study population and data source

**Figure 1:**
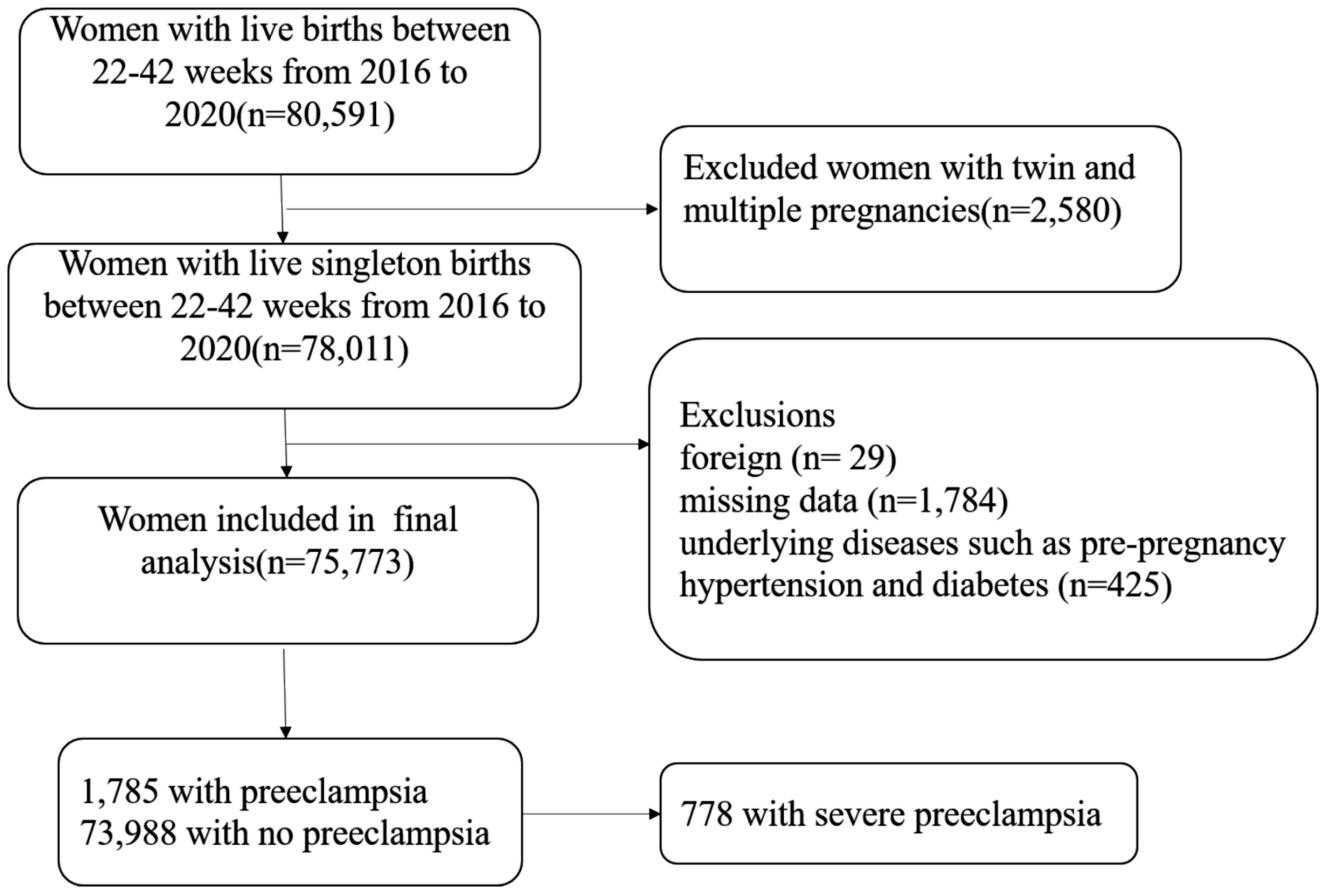
Illustration of the participant recruitment process.

The investigation was initiated at Shanghai First Maternity and Infant Hospital in China. The inclusion criteria were as follows: (1) Participants had a live birth of a single infant; (2) participants must have delivered between 22nd and 42nd week of gestation; (3) participants must have given birth at Shanghai First Maternity and Infant Hospital in China; (4) delivery records must have been documented between the years 2016 and 2020; and (5) delivery records must be accessible in the hospital’s electronic medical system. Participants with records meeting the following criteria were excluded: (1) Twin or multiple pregnancies; (2) foreign nationality; (3) incomplete clinical information; and (4) underlying diseases such as pre-pregnancy hypertension and diabetes. Among the recruited participants, 73,988 individuals did not have preeclampsia, while 1,785 were diagnosed with preeclampsia, including 778 cases classified as severe preeclampsia. (Figure 1). The research was approved by the Ethics Committee of Shanghai First Maternity and Infant Hospital, School of Medicine, Tongji University.

Personal health and sociodemographic information was collected during outpatient consultation, including maternal age, parity status (nulliparous/multiparous), whether conception was through assisted reproductive technology (ART), native place, nationality, employment status, maternal height, and maternal weight before pregnancy. The value of BMI was computed as weight (in kilograms)/height (in meters squared) and classified as underweight (<18.5 kg/m^2^), optimal weight (18.5–23 kg/m^2^), overweight (23–27.5 kg/m^2^), and obesity (≥27.5 kg/m^2^), based on the criteria suitable for the Asian population. Gestational age was determined based on the date of the last menstrual period. Information about the delivery mode and newborn status was extracted from the electronic system.

### Diagnosis of preeclampsia and severe preeclampsia

Diagnosis of (severe) preeclampsia was based on the patient information documented in discharge records. The diagnosis criteria followed the guidelines by the Société Française d’Anesthésie et de Réanimation (3). Preeclampsia is defined as the onset of systolic blood pressure (SBP) ≥ 140 and/or diastolic blood pressure (DBP) ≥ 90 mmHg after the 20th week of gestation, along with proteinuria ≥0.3 g/24 h. Severe preeclampsia is diagnosed based on the presence of more than one of the following criteria: (1) Thrombocytopenia with a platelet count <100,000/mm^3^; (2) proteinuria >3 g/24 h; (3) neurological symptoms; (4) shortness of breath, acute pulmonary edema, or chest pain; (5) oliguria ≤500 mL/24 h or ≤ 25 mL/h; (6) uncontrolled hypertension with continuing DBP ≥ 110 mmHg or SBP ≥ 160 mmHg; (7) cytolysis in hepatic cells with AST/ALT levels greater than twice the normal limit, persistently; (8) persistent or severe upper abdominal pain, particularly in the right upper quadrant; and (9) serum creatinine ≥90 μmol/L.

### Statistical analyses

The χ^2^ test was applied to evaluate the associations between categorical variables with sufficient expected frequencies. To assess non-linear relationships between the pre-pregnancy BMI levels and (severe) preeclampsia outcomes while controlling for confounding variables, a logistic regression model was applied. The categorical confounders were defined based on prior references. The only factors found to be clinically significant in the univariate logistic regression analysis were adjusted for in the resulting model. All data processing and analyses were conducted using IBM SPSS Statistics, Version 26.0 (Armonk, NY: IBM Corp.). A significance level of *p* < 0.05 was applied for all statistical tests.

## Results

As illustrated in Table 1, the enrolled pregnant women were categorized by the BMI levels before pregnancy. The underweight cohort comprised 11,360 individuals with BMI values <18.5 kg/m^2^, the normal weight cohort comprised 53,912 individuals with BMI values ranging from 18.5 to <24 kg/m^2^, the overweight group comprised 8,756 individuals with BMI values ranging from 24 to <28 kg/m^2^, and the obese group comprised 1,745 individuals with BMI values ≥28 kg/m^2^.

**Table 1:** Information about the enrolled participants, divided based on pre-pregnancy BMI values.

	<18.5 (kg/m^2^)	18.5- < 24 (kg/m^2^)	24- < 28 (kg/m^2^)	≥28 (kg/m^2^)
Age group, years, n (%)				
<35	10,488(92.32)*	45,973(85.27)	6,892(78.71)*	1,425(81.66)*
≥35	872(7.68)	7,939(14.73)	1,864(21.29)	320(18.34)
Parity status, n (%)				
Nulliparous	9,396(82.71)*	40,449(75.03)	6,064(69.26)*	1,220(69.91)*
Multiparous	1,964(17.29)	13,463(24.97)	2,692(30.74)	525(30.09)
Mode of delivery, n (%)				
Vaginal delivery	8,557(75.33)*	36,798(68.26)	4,836(55.23)*	867(49.68)*
Cesarean section	2,803(24.67)	17,114(31.74)	3,920(44.77)	878(50.32)
Assisted reproductive technology, n (%)				
Yes	434(3.82)*	3,260(6.05)	870(9.94)*	236(13.52)*
No	10,926(96.18)	50,652(93.95)	7,886(90.06)	1,509(86.48)
Native place, n (%)				
Shang Hai	3,439(30.27)	15,922(29.53)	3,185(36.38)*	791(45.33)*
Other areas	7,921(69.73)	37,990(70.47)	5,571(63.62)	954(54.67)
Nationality, n (%)				
Han	11,099(97.70)	52,584(97.54)	8,531(97.43)	1,698(97.31)
Other	261(2.30)	1,328(2.46)	225(2.57)	47(2.69)
Employment status, n (%)				
Yes	10,294(90.62)*	49,945(92.64)	8,048(91.91)*	1,594(91.35)*
No	1,066(9.38)	3,967(7.36)	708(8.09)	151(8.65)
Maternal height, centimeter				
<155	572(5.04)*	3,118(5.78)	579(6.61)*	108(6.19)*
155- < 165	7,790(68.57)	36,298(67.33)	5,841(66.71)	1,097(62.87)
≥165	2,998(26.39)	14,496(26.89)	2,336(26.68)	540(30.95)
Gestational diabetes mellitus, n (%)				
Yes	740(6.51)*	5,689(10.55)	1738(19.85)*	482(27.62)*
No	10,620(93.49)	48,223(89.45)	7,018(80.15)	1,263(72.38)

^*^p < 0.05. BMI, body mass index.

Compared to the normal BMI cohort, the overweight and obese cohorts exhibited a higher proportion of individuals aged 35 years and older (14.73% vs. 21.29 and 18.34%). In contrast, the underweight individuals were less likely to be of advanced maternal age (14.73% vs. 7.68%). The comparisons mentioned above regarding age showed statistically significant differences (*p <* 0.05). In addition, the overweight and obese populations showed a higher likelihood of being multiparous (24.97% vs. 30.74 and 30.09%), undergoing a cesarean delivery (31.74% vs. 44.77 and 50.32%), conceiving through assisted reproductive technology (ART) (6.05% vs. 9.94 and 13.52%), having a residence in Shanghai (29.53% vs. 36.38% and. 45.33%), and experiencing gestational diabetes mellitus (10.55% vs. 19.85% and. 27.62%), all of which were statistically significant. On the other hand, the underweight population was more likely to be primiparous (24.97% vs. 17.29%), received a cesarean delivery (31.74% vs. 24.67%), and conceived through ART (6.05% vs. 3.82%), all of which were also statistically significant.

Compared to the women with normal weight, the underweight pregnant women showed a significantly lower incidence of preeclampsia (1.27% vs. 2.08%), while the overweight and obese women exhibited notably higher preeclampsia rates, at 4.49 and 7.22%, respectively. Similarly, for severe preeclampsia, the incidence was significantly lower in the underweight women (0.67% vs. 0.96%), while the overweight and obese women exhibited significantly higher rates, at 1.59 and 2.58%, respectively, compared to the normal BMI group (Table 2).

**Table 2:** Relationship between the BMI values before pregnancy and (severe) preeclampsia in the singleton pregnant women.

	<18.5 (kg/m^2^)	18.5- < 24 (kg/m^2^)	24- < 28 (kg/m^2^)	≥28 (kg/m^2^)
Preeclampsia, n (%)	144(1.27)*	1,122(2.08)	393(4.49)*	126(7.22)*
Severe Preeclampsia, n (%)	76(0.67)*	518(0.96)	139(1.59)*	45(2.58)*

^*^p < 0.05. BMI, body mass index.

Logistic regression was applied to explore the relationship between BMI before pregnancy and the risks of preeclampsia and severe preeclampsia. Overweight and obese populations exhibited a higher risk of preeclampsia, with ORs (95% CI) of 2.211 (1.967–2.486) and 3.662 (3.026–4.431), respectively. In contrast, the individuals with a BMI value <18.5 kg/m^2^ exhibited a lower risk of preeclampsia, with an OR (95% CI) of 0.604 (0.507–0.719). After adjusting for confounders, including maternal age, gestational diabetes mellitus, parity, assisted reproductive technology, and employment status, these risks remained significant, with ORs (95% CI) of 2.152(1.911–2.425) for overweight women, 3.493(2.874–4.245) for obese women, and 0.609(0.511–0.727) for underweight women. All these differences were statistically significant. For severe preeclampsia, after adjusting for confounders, overweight and obese women still exhibited higher risks, with ORs (95% CI) of 1.652(1.364–2.001) and 2.762(2.014–3.788), respectively, while the underweight population exhibited a lower risk, with an OR (95% CI) of 0.720(0.565–0.919) (Table 3).

**Table 3:** Crude and adjusted ORs (95% CI) for the relationship between BMI before pregnancy and (severe) preeclampsia.

	<18.5 (kg/m^2^)	18.5- < 24 (kg/m^2^)	24- < 28 (kg/m^2^)	≥28 (kg/m^2^)
Preeclampsia				
Crude OR	0.604(0.507–0.719)*	1(Reference)	2.211(1.967–2.486)*	3.662(3.026–4.431)*
Adjusted OR^a^	0.609(0.511–0.727)*	1(Reference)	2.152(1.911–2.425)*	3.493(2.874–4.245)*
Severe preeclampsia				
Crude OR	0.694(0.545–0.884)*	1(Reference)	1.814(1.502–2.190)*	3.273(2.403–4.459)*
Adjusted OR^a^	0.720(0.565–0.919)*	1(Reference)	1.652(1.364–2.001)*	2.762(2.014–3.788)*

a Adjusted for gestational diabetes mellitus, maternal age, parity (nulliparous, multiparous), assisted reproductive technology, and employment status.

BMI, body mass index; OR, odds ratio; CI, confidence interval. *p < 0.05.

The women were stratified by age to explore whether the relationship between BMI before pregnancy and the risks of preeclampsia and severe preeclampsia varied across different age groups. Among the women younger than 35 years, after adjusting for confounders, those with a BMI value <18.5 kg/m^2^ exhibited a lower risk of preeclampsia (OR = 0.588, 95% CI =0.487–0.708), while the overweight (OR = 2.119, 95% CI =1.852–2.424) and obese (OR = 3.751, 95% CI = 3.035–4.635) women exhibited higher risks. For severe preeclampsia, the underweight population showed a lower risk (OR = 0.739, 95% CI = 0.573–0.954), while the overweight (OR = 1.605, 95% CI = 1.285–2.003) and obese (OR = 2.890, 95% CI = 2.039–4.095) women still showed an elevated risk. Among women aged 35 years or older, there was no reduction in the risk of preeclampsia or severe preeclampsia in underweight women compared to those with a normal BMI. However, the risk was higher for overweight and obese women (Table 4). In addition, these risks were not significantly associated with maternal age.

**Table 4:** Crude and adjusted ORs (95% CI) for the relationship between BMI before pregnancy and (severe) preeclampsia categorized by age.

	<18.5 (kg/m^2^)	18.5- < 24 (kg/m^2^)	24- < 28 (kg/m^2^)	≥28 (kg/m^2^)
<35, y				
Preeclampsia				
Crude OR	0.587(0.487–0.707)*	1(Reference)	2.139(1.873–2.443)*	3.895(3.168–4.788)*
Adjusted OR^a^	0.588(0.487–0.708)*	1(Reference)	2.119(1.852–2.424)*	3.751(3.035–4.635)*
Severe preeclampsia				
Crude OR	0.725(0.563–0.935)*	1(Reference)	1.707(1.371–2.127)*	3.376(2.401–4.748)*
Adjusted OR^a^	0.739(0.573–0.954)*	1(Reference)	1.605(1.285–2.003)*	2.890(2.039–4.095)*
≥35, y				
Preeclampsia				
Crude OR	0.834(0.497–1.399)	1(Reference)	2.423(1.879–3.125)*	2.660(1.615–4.380)*
Adjusted OR^a^	0.831(0.494–1.398)	1(Reference)	2.296(1.774–2.972)*	2.455(1.480–4.072)*
Severe preeclampsia				
Crude OR	0.566(0.247–1.295)	1(Reference)	2.019(1.386–2.941)*	2.793(1.342–5.813)*
Adjusted OR^a^	0.567(0.247–1.301)	1(Reference)	1.835(1.251–2.693)*	2.307(1.092–4.875)*

a Adjusted for gestational diabetes mellitus, maternal age, parity (nulliparous, multiparous), assisted reproductive technology, and employment status.

BMI, body mass index; OR, odds ratio; CI, confidence interval. *p < 0.05.

## Discussion

As we know, this is currently the largest retrospective study in China investigating the relationship between pre-pregnancy BMI and preeclampsia, as well as severe preeclampsia. In this study, the pregnant women who were overweight or obese before pregnancy were observed to have a higher risk of (severe) preeclampsia, while those who were underweight had a lower risk of developing preeclampsia. In addition, these risks were not significantly associated with maternal age.

Maternal obesity has been identified as an established risk factor for preeclampsia (15). The aforementioned positive connection between overweight/obesity before pregnancy and preeclampsia has been well-established (20–23). This research further confirmed that this correlation persists regardless of age or parity. The significance of the relationship became even more significant after excluding the effects of the factors mentioned above, as maternal overweight highly correlates with advanced age and multiparity (24–26). The risk of preeclampsia with serious complications was also increased in the overweight and obese populations and decreased in the underweight population. After adjusting for confounding factors, these correlations became even more significant. The underlying mechanisms still need further investigation, and it is hypothesized that both the initiation and progression of preeclampsia are influenced by similar adverse environmental factors in the overweight population.

There is evidence supporting the relationship between overweight and obesity and an increased risk of preeclampsia, especially its severe form. The onset of preeclampsia is characterized by the activation of endothelial cells, intravascular inflammation, and stress in the syncytiotrophoblast. These conditions are often triggered by a physiological environment in individuals with metabolic abnormalities, which are marked by systemic inflammation, increased oxidative stress, and endothelial dysfunction. Another possibility is that preeclampsia and metabolic abnormalities (including overweight and obese) share multiple co-activation pathways (27). This association is particularly pronounced in late-onset severe preeclampsia (28) as many manifestations of severe preeclampsia are associated with a highly inflammatory and hyper-reactive internal environment (29). As a clinically complex syndrome with multifactorial causes, the initiation and progression of preeclampsia involve various etiological factors.

Among multi-organ severe damage caused by preeclampsia across various systemic functions, conditions such as renal impairment, thrombocytopenia, and cardiopulmonary symptoms show a typical relationship with obesity-induced endothelial dysfunction. Excessive release of adipokines and inflammatory cytokines from the adipose tissue contributes to the chronic inflammatory state, damaging endothelial cells and affecting coagulation mechanisms. This is associated with vascular lesions and impairment in the regulation of blood pressure. Hypertension and systemic endothelial injury ultimately lead to end-organ dysfunction. This condition is also linked to short-term symptoms of preeclampsia, such as headache, blurred vision, and photophobia (30). Moreover, abnormal fatty acid metabolism is another issue commonly seen in the overweight population as it contributes to placental oxidative stress (31) and modulates the balance between thromboxane and prostacyclin (32). Therefore, the disruption of long-chain polyunsaturated fatty acids, which is commonly observed in the overweight or obese population, may contribute to the onset of preeclampsia (33). However, since this was a retrospective study and lacked sufficiently detailed information on lipid metabolism, we could not be certain if it is related to this factor. Furthermore, obesity increases the overall fluid load and renal burden, leading to altered renal blood perfusion. This ultimately causes or enhances kidney injury, which is manifested as elevated serum creatinine levels. Concurrently, renal dysfunction is a critical hallmark of severe preeclampsia. Obesity not only amplifies cardiac load, leading to cardiopulmonary symptoms, but also poses a risk of inducing structural and functional heart alterations over the long term, which, in turn, can elevate the risk of heart failure. Overall, the complex pathogenic pathways in pre-pregnancy overweight or obese population create a conducive foundation for the onset and progression of preeclampsia, thereby strengthening the theoretical basis for our analysis and conclusions.

Therefore, it can be concluded that optimal weight management before conception is crucial in mitigating the risks associated with preeclampsia. Such practices would not only help lower the incidence of preeclampsia but also reduce the likelihood of developing its severe forms, which are characterized by life-threatening complications. Preeclampsia is a prevalent obstetric complication, with an incidence rate as high as 2.2–9.4% in China (21, 34, 35). It is the leading cause of maternal death globally, second only to postpartum hemorrhage, and impacts the health of both the fetus and mother in the short term and long term, making it a critical issue in obstetrics. By managing weight, the pathophysiological factors that contribute to the development of preeclampsia can be more effectively moderated. Consequently, this approach leads to a reduction in the severity and incidence of preeclampsia, thereby enhancing the safety and viability of pregnancies. This study still has several limitations. As a retrospective analysis, all the data used were sourced only from the electronic register system. The lack of records regarding prior pregnancies with preeclampsia, placental abnormalities, weight gain during pregnancy, fetal gender, autoimmune and kidney diseases, socioeconomic status, weight loss treatments or relevant medication use, and the pregnancy stage at which preeclampsia was diagnosed makes it difficult to rule out these confounding factors or perform a detailed stratification of preeclampsia into early- or late-onset categories. Moreover, the absence of information on the specific complication of each patient diagnosed with severe preeclampsia limited the discussion on the role that pre-pregnancy BMI plays in different situations, such as dysfunction occurring in various organs, including hepatic, renal, and blood pressure regulation disorders.

## Conclusion

Our study demonstrated that pre-pregnancy underweight individuals exhibited a significantly lower chance of developing preeclampsia, irrespective of confounding factors, while pre-pregnancy overweight and obese individuals exhibited an elevated risk for both preeclampsia and severe preeclampsia. The results showed no correlation with maternal age, suggesting that BMI before pregnancy is a critical determinant in assessing the risk of preeclampsia. The findings reveal the significance of weight management before pregnancy in reducing the incidence of preeclampsia and its severe forms.

## Data Availability

The raw data supporting the conclusions of this article will be made available by the authors, upon reasonable request. Requests to access these datasets should be directed to 2565484846@qq.com.
